# Microbiological Foundations to Optimise Intrinsic Capacity and Promote Healthy Ageing: An Integration Into the Life Course Approach

**DOI:** 10.1111/acel.70146

**Published:** 2025-06-27

**Authors:** Christoph Benner, Matteo Cesari, Ritu Sadana

**Affiliations:** ^1^ Ageing and Health Unit, Department of Maternal, Newborn, Child, and Adolescent Health and Ageing World Health Organization Geneva Switzerland

**Keywords:** ageing, biology, nutrition, prevention

## Abstract

The World Health Organization (WHO) defines healthy ageing as the process of developing and maintaining functional ability, comprising an individual's intrinsic capacity, the environment and the interaction of the two. The framework is based on a positive approach to ageing, giving value to the resources individuals can rely upon as they age and that they can build their physical, mental and social health, and overall well‐being. To promote healthy ageing, it is important to understand better the biological mechanisms underlying this phenomenon from this positive perspective. Our knowledge about cellular processes that drive human ageing has increased dramatically, with current evidence identifying 12 hallmarks of ageing. Dysbiosis is one of these and is broadly defined as a ‘deranged microbiological composition in and on the human body’. It is often measured by quantitatively and qualitatively evaluating the bacterial species in the gut. A major feature of dysbiosis and other markers of ageing is that these focus on age‐related impairments, contributing to the onset of adverse outcomes over time rather than highlighting features that promote healthy ageing. Scientific literature addressing the hallmarks of healthy ageing, including those potentially positively affecting intrinsic capacity, is lacking. To this end, we propose the concept of gut eubiosis, the homeostatic state of commensal gut bacteria and their metabolites, as proof of concept, serving as a hallmark of healthy ageing. Importantly, this work adopts a life course approach to explore how a person's intrinsic capacities evolve with gut microbiota modifications at different life stages.

## Graphical abstract

Gut microbiota composition may influence trajectories of intrinsic capacity throughout life. Factors that positively or negatively impact gut microbiota (determining eubiosis or dysbiosis, respectively) can affect health, potentially acting through short‐chain fatty acids and their metabolic and anti‐inflammatory functions. Created in BioRender.com (https://BioRender.com/h4sp9g2).

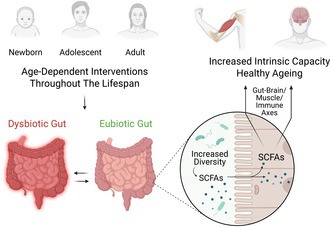

## 1 Introduction

### 1.1 Population Ageing, Healthy Life Expectancy and Healthy Ageing

In 2020, the global population aged 60 and over surpassed 1 billion, accounting for more than 13.5% of the world's population. According to the WHO, forecasts indicate that the number of individuals in this age group is expected to rapidly double, reaching approximately 2.1 billion (i.e., 20% of the world population) by 2050 (World Health Organization 2020). This reflects the increase in global life expectancy (LE) and the reduction of fertility rates. However, extending LE does not necessarily mean people live in good health. Evidence shows that, on average, the last 10%–15% of an individual's life (13%–16% for women and 11%–12% for men) is spent in ill health with one or more morbidities, including chronic diseases and declines in physical and mental capacities. A primary goal of population health policies is, therefore, to promote healthy ageing, preventing the clinical and social conditions impacting the ability of the individual to live a long, independent and fulfilling life.

The WHO (World Health Organization 2017) defines healthy ageing as the process of developing and maintaining functional ability for well‐being in older age. Functional ability reflects a person's intrinsic capacity, the environment (extrinsic factors surrounding the person, including social values, policies and access to programmes and services in health and other sectors) and the interactions of these two. Intrinsic capacity is the composite of an individual's physical and mental capacities, organised around the locomotion, cognition, psychology, vitality and sensory (i.e., hearing and vision) domains (Cesari et al. 2018). The constituent domains of intrinsic capacity were initially derived from a literature review adopting the International Classification of Functioning, Disability and Health (ICF) as the operational framework. In particular, the domains of interest were identified from the body functions that, when impaired, were associated with an increased risk of incident disability in older persons. By focusing on body functions, the intrinsic capacity construct implicitly promotes a life course approach based on the biology of the individual, better reflecting the experience of ageing compared to traditional models based on age or diseases. Indeed, it emphasises a life course perspective, recognising the importance of each life stage and the intergenerational relationships in developing and maintaining health throughout life (World Health Organization 2017; United Nations 2020). Importantly, each of the domains of intrinsic capacity can be assessed and quantified by medical and scientific measures. In fact, their impairments are phenotypically expressed by conditions of risk that are frequently observed (and neglected) in clinical practice: mobility limitation, cognitive impairment, depressive symptoms, undernutrition, hearing loss and vision impairment. This opens the possibility of combining the WHO's definition of healthy ageing with scientifically defined markers of ageing and clinically meaningful markers to improve care for older persons. This symbiosis is largely understudied but may provide levers to adopt more effective global health policies.

It is noteworthy that constructs other than intrinsic capacity exist in research and clinical activities to provide surrogates of biological ageing, for example, frailty (i.e., a medical condition characterised by enhanced vulnerability to stressors due to a reduction of homeostatic reserves, exposing the individual to increased risk of adverse health outcomes) (Morley et al. 2013). Despite frailty and intrinsic capacity share several commonalities, they also differ in several perspectives (Belloni and Cesari 2019). Both were conceived to promote a different approach to the complexity of older persons and ensure tailored care according to their heterogeneous needs. However, whereas frailty focuses on capturing the individual's health deficits using diverse operational instruments, intrinsic capacity derives from a public health framework prioritising the person's capacities and reserves, also to promote a positive vision of ageing. Moreover, frailty may represent the target condition for the work (e.g., comprehensive geriatric assessment) conducted by geriatricians in their specialistic settings (Ellis et al. 2017); differently, intrinsic capacity is a construct designed to promote a different approach to older persons across settings of care (in particular, in primary health care) and among health and care workers (World Health Organization 2024). An additional major difference between the two constructs is also the predefined operational model of intrinsic capacity (i.e., its constituent domains), allowing a standardisation of the approach that is currently hampered by the different translations that the frailty concept has undergone over the years (Cesari et al. 2022; Aguayo et al. 2017).

### 1.2 A Hallmark of Healthy Ageing: From Dysbiosis to Eubiosis

Numerous scientific studies have shown that various positive and negative factors can influence the trajectory of ageing. Lifestyle choices, behaviours and opportunities, such as regular exercise and dietary strategies (e.g., caloric restriction), have beneficial effects on health and longevity in both animal and human models. At the same time, a wide range of research demonstrates that these results are associated with parallel improvements of the so‐called ‘hallmarks of ageing’ (López‐Otín et al. 2023). These hallmarks include genetic instability, telomere attrition, epigenetic alteration, loss of proteostasis, disabled macroautophagy, deregulated nutrient‐sensing, mitochondrial dysfunction, cellular senescence, stem cell exhaustion, altered intercellular communication, chronic inflammation and dysbiosis.

Among these hallmarks, dysbiosis stands out as a recently identified factor (as compared to those presented in the first landmark publication (López‐Otín et al. 2013) and has drawn much attention in the scientific and medical community over the last decade (Marchesi et al. 2016). Dysbiosis refers to the imbalanced composition of microbiological species in and on our body (i.e., microbiota) and the total number of genes they contain (i.e., microbiome). A dysbiotic state reflects a higher propensity towards unhealthy ageing. In contrast, eubiosis, the condition of homeostatic balance of microbiota, can be conceptualised as a state of higher propensity towards *healthy* ageing. Most of the research in the field has particularly focused on the approximately 2000 bacterial species in the gut and their relationships with several common diseases and conditions, such as obesity (Van Hul and Cani 2023), type 2 diabetes (Iatcu et al. 2021) or irritable bowel syndrome (Ghaffari et al. 2022).

To investigate how eubiosis may contribute to healthy ageing, it is necessary to accept the intricate relationship between bacteria and their human hosts. It has been discussed that the total number of bacteria outnumbers the cells that make up the human body (Berg 1996) (although these statements have been recently argued (Sender et al. 2016)) and that the number of non‐redundant genes within the microbiota exceeds the number of human genes by approximately 150 times (Zhu et al. 2010). Fascinatingly, gut microbiota has been indicated as a ‘virtual endocrine organ’ (Clarke et al. 2014), potentially contributing to an individual's overall health status. Some bacterial‐derived chemicals influence behavioural traits, such as food preference, via the gut–brain axis (Morais et al. 2021). This intimate relationship between bacteria and their human hosts has been developing into the notion of humans as holobionts, a functional unit of life comprising a symbiosis of eukaryotic and prokaryotic cells (van de Guchte et al. 2018).

### 1.3 Objectives and Approach

**FIGURE 1:**
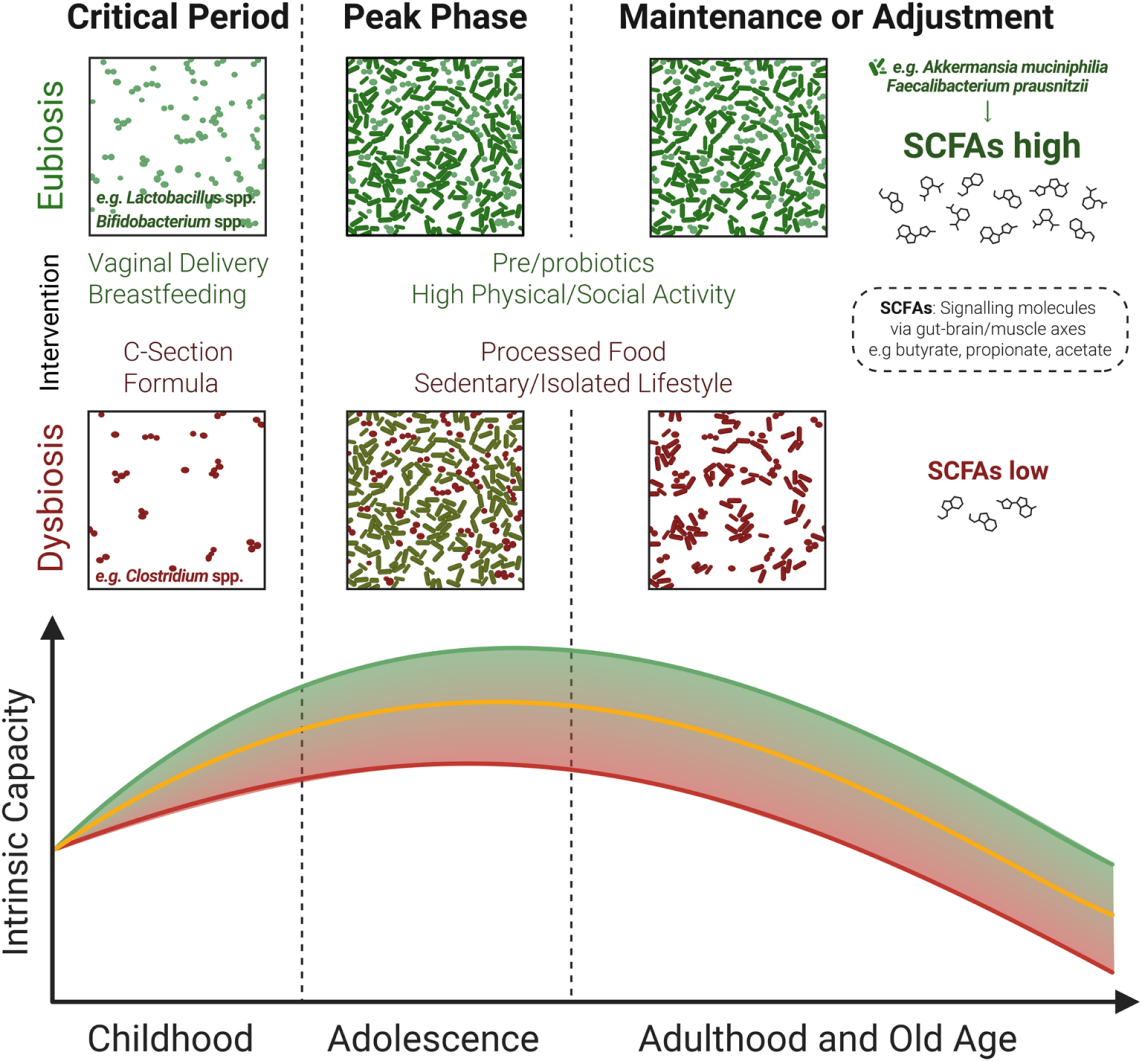
Association between gut microbiota and the trajectory of intrinsic capacity over a life course. The figure illustrates the dynamic interplay between gut microbiota states (i.e., eubiosis and dysbiosis) and intrinsic capacity across life stages. Throughout life, positive factors that potentially promote eubiosis (green; e.g., vaginal delivery, breastfeeding, immune system stimulation, engaging environment) may support the development and maintenance of higher trajectories of intrinsic capacity. Differently, dysbiosis (red), associated with factors such as C‐section delivery, formula feeding and poor lifestyle, may result in lower concentrations of short‐chain fatty acids (SCFAs) and poorer trajectories of intrinsic capacity. It is noteworthy that exposure to a factor does not necessarily determine the subsequent trajectory, as this latter results from potentially multiple, simultaneous, interacting and counteracting stimuli occurring throughout life. Created using elements from BioRender.com.

This article explores and summarises the available scientific literature on the connection between gut microbiota and intrinsic capacity. In particular, we are interested in understanding how an eubiotic gut is defined and shaped across life stages in the development of healthy ageing. As depicted in Figure 1, we focus on three main features connecting eubiosis and intrinsic capacity:

1. Critical events in very early life (roughly until 2 years of age) determine the initial colonisation of gut bacteria and their relative abundance. These bacterial signatures contribute to immediate and later life health outcomes, especially affecting the vitality domain of intrinsic capacity.
2. Especially in adults, bacterial metabolites called short‐chain fatty acids (SCFAs) are positively correlated with intrinsic capacity domains locomotion and vitality. Studies also indicate alpha diversity (i.e., the variety of species and composition within an individual) to be a contributing factor, but to a lesser extent.
3. Lifestyle changes and opportunities (e.g., diet and physical activity) can easily modify these features of gut microbiota composition, providing a potentially important intervention to improve intrinsic capacity or prevent functional decline.

To this end, a purposive literature review was conducted using PubMed. In particular, evidence was retrieved from studies conducted in the general population, and an approach was adopted that was consistent with the WHO framework of healthy ageing. Publications were included if they investigated the association between specific gut microbiota features (i.e., alpha diversity and/or SCFA content) and one of the five domains of intrinsic capacity. Studies based on clinical settings were excluded to reduce the risk that index conditions could potentially introduce a bias in the gut microbiota profile, the domains of intrinsic capacity or the relationship between the two.

## 2 Findings

### 2.1 Critical Periods Determining the Formation of an Eubiotic Gut

#### 2.1.1 Birth, Infants and Children

The first crucial encounter between a newborn and bacteria occurs during delivery. The way of delivery, vaginal versus caesarean (‘C‐section’), provides the newborn with different sets of bacteria. As depicted in Figure 1, this is already an essential distinguishing feature concerning immediate and subsequent health trajectories. Vaginal delivery is known to provide bacteria from the genus *Lactobacillus* to the skin and mouth of the neonate. In contrast, the C‐section provides bacteria associated with maternal skin and the hospital environment, such as *Clostridioides difficile* and *Staphylococcus* spp. (Coelho et al. 2021). Gut colonisation with *Staphylococcus aureus* is relatively common, observed in up to 20% of healthy individuals and is not inherently pathogenic (Raineri et al. 2022). However, in certain contexts such as prematurity or immune immaturity, gut colonisation with *Staphylococcus* spp. has been associated with an elevated risk of invasive infections, including neonatal sepsis (Schwartz et al. 2023). Another key characteristic of babies born via C‐section and the related intrapartum use of antibiotics is depletion of *Bacteroides* spp. (Pivrncova et al. 2022). Several studies have linked the bacterial signature associated with caesarean delivery to a higher risk of developing metabolic disorders affecting the vitality domain (e.g., obesity, type 2 diabetes) and immune dysfunctions (e.g., asthma, juvenile arthritis, food allergy) at later developmental stages (Słabuszewska‐Jóźwiak et al. 2020; Sevelsted et al. 2015; Zhang et al. 2021; Yang et al. 2022; Chavarro et al. 2020). To adjust for a missed health‐promoting opportunity, the practice of vaginal seeding (i.e., exposing C‐section‐delivered infants to maternal vaginal microbiota) has been explored in research settings especially given the growing number of C‐section deliveries, often conducted without medical necessity (Angolile et al. 2023). Initial pilot studies have provided promising, but partial, evidence that vaginal microbial transfer can alter the infant microbiome to more closely resemble that of vaginally delivered infants (Dominguez‐Bello et al. 2016). However, further studies have shown mixed results regarding the efficacy of vaginal seeding in restoring the microbiome of C‐section‐born infants, particularly the *Bacteroides* signature (Dos Santos et al. 2023; Wilson et al. 2021). Currently, major professional societies, such as the American College of Obstetricians and Gynaecologists (ACOG), do not recommend or encourage vaginal seeding outside of controlled research protocols, primarily citing concerns about safety and insufficient evidence of benefit (Anon 2017).

Another important driving force behind the establishment of the gut microbiota is the type of milk used as a primary food source (i.e., maternal breast milk vs. formula milk). Irrespective of the way of feeding, *Bifidobacterium* spp. is one of the most predominant bacteria that colonise the gut after feeding initiation (Saturio et al. 2021), as these are responsible for the fermentation of galacto‐oligosaccharides. However, breast milk feeding supplies infants with a more diverse set of *Bifidobacterium* spp. and other beneficial bacterial species, along with immune cells and stem cells, all of which positively contribute to neonatal health (Saturio et al. 2021; Henrick et al. 2021; Ninkina et al. 2019; Lokossou et al. 2022). Breastfeeding is also known to exert protective effects against diarrhoea during the first months after delivery (Santos et al. 2015) and produce later benefits by reducing the risk of incident obesity (Qiao et al. 2020; Li et al. 2022) and asthma (Xue et al. 2021). Of note, a recent review analysed the effect of breastfeeding on the gut microbiota of C‐section delivered infants during their first 3 months (Pivrncova et al. 2022). The authors concluded that while breastfeeding cannot reverse the reduced abundance of *Bacteroidetes* associated with C‐section and antibiotic exposure, it does have a positive effect on increasing *Bifidobacterium* spp. This is also supported by results of another publication showing that exclusive breastfeeding following caesarean delivery partially restores microbiota diversity and composition, particularly increasing *Bifidobacterium* spp., and is associated with a reduced incidence of respiratory infections in infancy (Liu et al. 2023). A similar result was shown in infants exclusively fed either breast milk (BF) or formula (FF) for at least 4 months. They were delivered via either vaginal delivery (VD) or caesarean section (CS). At 40 days, *Bifidobacteria* were significantly more abundant in the CS‐BF group compared to the CS‐FF group. No significant differences were observed between the VD‐BF and VD‐FF groups. By 3 and 6 months, *Bifidobacterium* spp. levels converged across all groups. In addition, the CS‐FF group had higher abundances of *Enterococcus* and *Streptococcus* compared to breastfed groups. By 3 months, *Enterococcus* spp. levels remained elevated in formula‐fed infants, regardless of delivery model (Ma et al. 2022). This nuanced comparison supports a gradient model where early microbial environments can enhance or diminish intrinsic capacity reserves. In addition, these studies underscore the complexity of neonatal gut microbiome development and suggest that exclusive breastfeeding after vaginal delivery most positively influences gut microbiota composition. In addition, supplementation with probiotics, prebiotics or synbiotics during pregnancy or lactation may be an effective treatment against this loss of gut microbiological biodiversity (Martín‐Peláez et al. 2022).

Other environmental factors acting on a newborn, infant or toddler may influence health outcomes later in life, including the use of probiotics, the presence of pets, the level of hygiene and, most directly, antibiotic treatments (Figure 1). From an initial low diversity and low complexity, the infant's intestinal microbiota will slowly develop and mature, reaching an adult‐like richness at around 3 years of age (Yatsunenko et al. 2012). Although matured, a child's microbiota composition during the first decade of life is still malleable. It provides a critical period to promote optimal development and healthy ageing through environmental factors, especially diet, as described in detail elsewhere (Derrien et al. 2019).

#### 2.1.2 Adolescence

Adolescence is defined as the developmental period between childhood and adulthood, approximately spanning 11–21 years of age (Hardin et al. 2017). Sexual maturation is one of the key physiological processes during this time. Concerning the gut microbiota, a sex‐specific connection between oestrogen and testosterone levels and variations in the composition and diversity of gut microbiota has been reported (d'Afflitto et al. 2022). In addition to sex hormone fluctuation, the brain undergoes fundamental changes during adolescence; the stress‐related hypothalamic–pituitary–adrenal axis matures to become highly responsive during adulthood (Lupien et al. 2009). This process makes this life stage highly susceptible to stress‐related psychiatric disorders (Paus et al. 2008).

A systematic review examined the relationship of the gut microbiota with cognition and anxiety during adolescence (Basso et al. 2022). Regarding cognition, 19 studies assessed cognitive outcomes, including 13 focusing on prebiotics as a possible treatment option. Using ‘psychobiotics’ as a therapeutic measure for cognitive enhancement during development appears promising, with half of the studies reporting positive results. However, the heterogeneity of study designs, interventions and participants hindered the potential for a comprehensive meta‐analysis. Additionally, the vast array of cognitive functions examined, which encompassed various aspects of attention, executive functions and working memory, further complicated the comparisons and definitive conclusions.

In the same review, 17 studies focused on anxiety, with 11 and six studies testing probiotic and prebiotic interventions, respectively. However, also in this case, the evidence supporting the efficacy of probiotic and prebiotic interventions in mitigating anxiety and stress in paediatric and adolescent populations remained limited for the same methodological reasons.

A similar complex scenario is drawn by systematic reviews reporting the association of gut microbiota with adiposity in adolescence (Vander Wyst et al. 2021; Morgado et al. 2023). Some studies reported the beneficial effects of physical exercise on alpha diversity, and positive results were also documented on the effectiveness of dietary interventions to increase SCFA‐producing bacteria (e.g., *Faecalibacterium prausnitzii* ). However, data are still limited, and it is premature to confirm such associations.

#### 2.1.3 Adults

A clear‐cut definition of a healthy ageing‐promoting gut microbiota composition in adults is hampered by the fact that the phylum‐to‐species‐level composition of any given individual is highly diverse and unique (Manor et al. 2020), depending on geographical location, ethnicity (Gaulke and Sharpton 2018; Suzuki and Worobey 2014), diet and availability of food sources (Zhang 2022; Wilson et al. 2020), level of physical activity (Dziewiecka et al. 2022) and the housing situation (e.g., community‐dwellers versus residents in care facilities (Claesson et al. 2012). Therefore, it seems more appropriate to look at core traits and activities that more likely and stably characterise a gut microbiota composition and role in the promotion of healthy ageing rather than the species‐level composition. The scientific literature gathered in this investigation about domains of intrinsic capacity suggests that an overall increased concentration of SCFAs and an overall increased gut microbiota alpha diversity (potentially an underlying prerequisite for the former) represent important determinants of healthy ageing, including those noted in the subsequent sections.

##### 2.1.3.1 Short‐Chain Fatty Acids (SCFAs) Promote an Eubiotic Gut Microbiota Composition

SCFAs are produced by certain bacterial species (e.g., *Faecalibacterium prausnitzii* , *Eubacterium rectale* , *Roseburia intestinalis* ) in response to metabolising undigestible complex dietary carbohydrates (e.g., soluble fibres present in nuts, seeds or legumes) (Fu et al. 2022). Other factors that increase SCFAs are probiotics, physical fitness and the density of community living (Figure 1 and Table 1). The most prominent SCFAs in the gut are butyrate, acetate and propionate (Portincasa et al. 2022), which possess many health‐promoting effects (Xiong et al. 2022). For instance, regarding the gut itself, these exert local anti‐inflammatory effects and, vice versa, decrease the richness of bacterial strains that produce proinflammatory metabolites (David et al. 2014; Meslier et al. 2020). Through the gut–brain axis (Dalile et al. 2019), SCFAs influence the feeling of hunger and satiety as well as a person's food preferences, promoting the intake of food that stimulates SCFA‐producing bacteria (Tremaroli and Bäckhed 2012). Also, SCFAs exert anti‐inflammatory effects on the brain by modulating microglial activity, protecting against neurodegenerative disorders and preserving cognitive capacity (Zhou et al. 2022; Solanki et al. 2023). As signalling molecules, SCFAs additionally affect muscle metabolism through the gut–muscle axis (Chew et al. 2022; Przewłócka et al. 2020), thus supporting the maintenance of physical performance and muscle strength (and assessed within the intrinsic capacity domains of vitality and locomotion), discussed further in the next section.

**TABLE 1:** Gut microbiota and domains of intrinsic capacity.

Study	Trial design	Study cohort	Intervention/follow‐up	Main results: Alpha diversity change	Main results: SCFA changes	Associated intrinsic capacity
Ghosh et al. (2020)	Randomised, single‐blind, controlled	612 older adults (65–79 years), Europe	Mediterranean Diet (MedDiet)/follow‐up after 12 months	Higher in MedDiet Group	Increase in SCFA‐producing bacteria, e.g., *Faecalibacterium prausnitzii*	Cognition and locomotion (hand grip strength, gait speed)
Lv et al. (2021)	Cross‐sectional	482 Menopausal Women (41–65 years), China	NA/no follow‐up	NA	Mendelian randomisation‐derived causative association of genetically driven butyrate synthesis and increased appendicular lean mass	Locomotion (muscle mass, sarcopaenia)
Sanna et al. (2019)	Observational	952 normo‐glycaemic individuals, Netherlands	NA/no follow‐up	NA	Genetic‐driven increase in gut production of the SCFA butyrate is associated with improved insulin response following an oral glucose test.	Vitality (glucose metabolism)
Estaki et al. (2016)	Observational	39 Young Adults (18–35 years), Location not specified	NA/no follow‐up	Higher in high fitness group compared to low fitness group	Increased concentration of butyrate and abundance of butyrate‐producing bacteria	Locomotion and vitality (physical fitness, VO_2_ max)
Scheiman et al. (2019)	Observational	15 Marathon runners, 10 sedentary controls	NA/no follow‐up	NA	*Veillonella* species in abundance in marathon runners, producing SCFAs	Locomotion (physical fitness and endurance)
Castro‐Mejía et al. (2020)	Cross‐sectional	207 Older Individuals (> 65 years), Denmark	NA/no follow‐up	Higher in high fitness group compared to low fitness group	No significant association to physical fitness	Locomotion (muscle strength), Vitality (metabolic health)
Huang et al. (2020)	Double‐blind, placebo controlled	20 Male Athletes, Taipei City University	Probiotic Supplementation/follow‐up after 6 weeks	Lower in probiotic group, but more SCFA‐producing bacteria	Increase in faecal SCFAs butyrate, propionate, acetate	Vitality (endurance capacity)

*Note:* Main studies showing the beneficial effects of increasing gut microbiota diversity and gut microbiota‐generated SCFAs concentrations on intrinsic capacity domains (in particular, locomotion, vitality and cognition). Included studies were selected based on outcomes that correspond to domains of intrinsic capacity as defined by the WHO, even if intrinsic capacity was not the stated framework of the original study.

##### 2.1.3.2 Association of Gut Microbiota Composition With Intrinsic Capacity and Frailty

*Intrinsic capacity*. Table 1 summarises our findings regarding the association of gut microbiota compositions and domains of intrinsic capacity. An intervention study recruiting 612 non‐ or pre‐frail older individuals from five European countries highlighted the benefits of a fibre‐rich Mediterranean diet (Ghosh et al. 2020). Adherence to this diet was linked to improved physical and cognitive outcomes, correlated with attenuated loss of microbiota diversity and a higher abundance of SCFA‐producing bacteria, which may enhance anti‐inflammatory markers while reducing pro‐inflammatory markers.

Research on menopausal Chinese women revealed a significant association between the gut microbiota's ability to produce butyrate and muscle mass (Lv et al. 2021), suggesting a protective role against sarcopaenia. The health‐promoting effect of butyrate is supported by findings from the Dutch Life‐Line Deep cohort, which linked butyrate production in the gut to improved metabolic responses in 952 normoglycaemic individuals (Sanna et al. 2019).

Data indicate a bidirectional relationship between physical activity and gut microbiota composition. Studies on both younger (Estaki et al. 2016) and older (Castro‐Mejía et al. 2020) individuals' document that physical fitness positively correlates with gut microbiota diversity and SCFA concentration. In support of that, *Veillonella* species are more abundant in marathon runners than in sedentary controls (Scheiman et al. 2019), as these metabolise lactate into the SCFAs acetate and propionate. Probiotic supplementation with a *Lactobacillus plantarum* strain was found to enhance physical fitness and muscle performance. Notably, this observation was associated with an overall lower alpha diversity but a higher amount of SCFA‐producing bacteria and higher acetate, propionate and butyrate production (Huang et al. 2020).

*Frailty*. The concept of intrinsic capacity shares many aspects with that of frailty (Belloni and Cesari 2019), a geriatric syndrome defined by the decline of the homeostatic capacities of the individual, responsible for an increased vulnerability to endogenous and exogenous stressors (Morley et al. 2013). The two concepts aim to capture the individual's homeostatic capacities, potentially supporting the estimate of the person's biological age. In this context, our review of the literature was secondarily extended to retrieve relevant evidence exploring the relationship between gut microbiota and frailty, which is summarised in Table 2.

**TABLE 2:** Gut microbiota and frailty.

Study	Trial design	Study cohort	Intervention/follow‐up	Main results: Alpha diversity change	Main results: SCFA changes	Frailty association
Ghosh et al. (2020)	Randomised, single‐blind, controlled	612 older adults (65–79 yo), Europe	Mediterranean Diet (MedDiet)/follow‐up after 6 months	Higher in MedDiet Group (associated with lower frailty)	Increase in SCFA‐producing bacteria, e.g., *Faecalibacterium prausnitzii* (associated with lower frailty)	Lower frailty
Jackson et al. (2016)	Cross‐sectional, observational	728 Female Twins (42–86 yo), UK	NA/no follow‐up	Lower diversity associated with higher frailty	Depletion of butyrate‐producing *Faecalibacterium prausnitzii* (associated with higher frailty)	Higher frailty
Haran et al. (2018)	Longitudinal (prospective)	23 Nursing Home Residents (≥ 65 yo), USA	NA/follow‐up after 6 months	NA	Decrease in butyrate‐producing bacteria (associated with higher frailty)	Higher frailty
Claesson et al. (2012)	Cross‐sectional, observational	178 Older Adults (64–102 yo), Ireland	NA/no follow‐up	Less diversity in long‐term care (associated with higher frailty)	NA	Higher frailty
Lim et al. (2021)	Cross‐sectional, observational	176 older persons (70–90 yo), Japan	NA/no follow‐up	NA	Negative association with frailty (butyrate‐producing *Coprococcus eutactus* associated with lower frailty)	Lower frailty

*Note:* Studies across different populations consistently linked a decrease in gut microbiota diversity and a reduction in SCFA‐producing bacteria, particularly butyrate producers, with increased frailty and poorer health outcomes.

Similar findings regarding the association between diet and frailty scores were observed in studies involving UK female twins (Jackson et al. 2016) and US nursing home residents (Haran et al. 2018), underscoring the negative impact of poor dietary intake of fibres on butyrate‐producing bacteria. Contrarily, community‐dwelling older persons exhibited healthier gut microbiota compositions than those in long‐term care institutions (Claesson et al. 2012), aspects that were explained as resulting from better diets and exercise regimens. A Japanese cohort study further affirmed the health‐promoting effects of butyrate‐producing bacteria on frailty (Lim et al. 2021).

Although frailty and intrinsic capacity are different concepts (i.e., frailty is defined by the individual's impairments and deficits, whereas intrinsic capacity focuses on the physical and mental capacities over the life course) their similarities might still allow us to obtain additional information to describe the clinical manifestations of the gut microbiota profile with ageing, relevant to potentially delaying or reversing declines.

Overall, these studies highlight the significant role of diet, gut microbiota and probiotic supplementation in promoting healthy ageing, muscle mass maintenance and physical capacity. They suggest potential interventions to counteract frailty and enhance overall health and intrinsic capacity in different population groups.

## 3 Discussion

To our knowledge, this article provides the first detailed description of the association between a hallmark of healthy ageing and intrinsic capacity, adopting a life course approach. Our overview of the literature shows that eubiosis can represent a critical biological feature that promotes healthy ageing by supporting the development and maintenance of intrinsic capacity, one of the key components of functional ability, across life stages. It may serve as a denominator to measure and monitor the health status of individuals as they age.

Incorporating the biological hallmarks of ageing into the framework of intrinsic capacity and its domain offers a pathway towards more personalised interventions, essential for effective health policies, care pathways and inclusion within universal health coverage packages. In this context, the potential of direct (e.g., faecal microbiota transplantation, FMT) and indirect (e.g., healthy diet) interventions to restore or enhance eubiosis is relevant, potentially offering a promising means to increase intrinsic capacity (in particular, its cognitive, locomotion, and vitality domains). For instance, small mechanistic trials show that transferring stool from lean donors can transiently improve peripheral insulin sensitivity in adults with metabolic syndrome (Kootte et al. 2017). A larger Chinese cohort study using ‘washed microbiota transplantation’ in 65 metabolic syndrome patients reported short‐ and medium‐term falls in fasting glucose and waist circumference after three courses of treatment, suggesting that protocol‐intensive FMT can hit several metabolic targets at once (Hu et al. 2024). A 2025 systematic review (Dhanasekaran et al. 2025) evaluated the efficacy of microbiome‐targeted interventions in obesity management, encompassing 27 randomised controlled trials. The review found that these interventions, including FMT, probiotics, prebiotics and synbiotics, led to significant improvements in body composition, metabolic parameters and inflammatory markers in overweight and obese adults. Notably, reductions in body mass index (BMI), fasting blood glucose and C‐reactive protein levels were observed, suggesting potential benefits in energy metabolism and overall vitality. In contrast, another recent meta‐analysis (Qiu et al. 2023), which included nine studies with a total of 303 participants, observed modest short‐term improvements in fasting blood glucose, HbA1c, insulin levels and HDL cholesterol following FMT. However, these benefits were not sustained beyond 6 weeks, and no significant changes were noted in weight or BMI. These conflicting findings highlight that, although studies provide promising insights into the role of microbiome‐targeted therapies in improving vitality and metabolic health, further research is needed to establish standardised protocols and long‐term efficacy.

By tailoring treatments to address specific biological changes associated with unhealthy ageing, health providers may offer more effective, individualised interventions targeting the underlying foundations of health deficits. At the same time, the biological mechanisms impacting intrinsic capacity domains may provide clear directions for policymakers in developing consistent preventive strategies. Promoting personalised interventions based on a specific biological substratum might be important to better address the heterogeneous needs of ageing populations. That most of these interventions could be provided across low‐, middle‐ and high‐income countries also increases opportunities for greater equity in healthy ageing.

Within the publications reviewed in this article, butyrate, a metabolic product of certain bacterial species, seemed to be the most important and consistent feature associated with increased intrinsic capacity and healthy ageing. In personalised medicine, butyrate levels could be tailored to individual needs, influenced by a person's unique gut microbiome composition, dietary habits and health status. Today, the functional capacity of the gut microbiota butyrate production can be monitored by standard laboratory equipment from readily available stool samples (Daskova et al. 2021), making its application in medicine and potential public health implementation a low hurdle. Furthermore, microbiota in personalised medicine is a promising new field (Ratiner et al. 2023), identified as an important contributor to drug metabolism and as an exploratory ground for individual variability in drug response (Zhao et al. 2023). Pharmaco‐microbiomics, the study of how the microbiota alters individual drug responses, might, therefore, stimulate the development of healthy ageing‐related treatment options.

Although butyrate exerts broad regulatory effects on host physiology, emerging evidence suggests that its influence is channelled through distinct anatomical and molecular host–microbe interaction axes (Morais et al. 2021; Fung 2020). This compartmentalised signalling likely contributes to domain‐specific effects on intrinsic capacity. The gut–muscle axis represents one of the most direct pathways through which butyrate supports intrinsic capacity, particularly within the vitality and locomotion domains. Butyrate has been shown to promote mitochondrial biogenesis and oxidative metabolism through activation of peroxisome proliferator‐activated receptor gamma coactivator 1‐alpha (PGC‐1α) and AMP‐activated protein kinase (AMPK) pathways (Gao et al. 2009). A recent review (Li et al. 2024) emphasises that gut microbiota dysbiosis impairs anabolic signalling, reduces insulin sensitivity and promotes muscle catabolism, highlighting the essential role of microbial‐derived SCFAs in muscle maintenance and sarcopaenia prevention (Li et al. 2024).

The gut–brain axis influences cognition by shaping microglial immune functions (Wang et al. 2018): Commensal microbes provide signals essential for microglial maturation and surveillance, e.g., germ‐free or antibiotic‐treated mice exhibit immature, hyper‐ramified microglia with impaired synaptic pruning and plasticity. Microbial SCFAs such as butyrate and propionate cross the blood–brain barrier to shift microglia towards an anti‐inflammatory, neurosupportive phenotype, thereby promoting neurogenesis and synaptic remodelling. Moreover, microbiota‐derived tryptophan metabolites acting via the aryl hydrocarbon receptor mitigate astrocyte‐driven inflammation and enhance trophic factor release, whereas dysbiosis‐induced peripheral cytokines (e.g., IL‐1β, TNF‐α) infiltrate the CNS, overactivate microglia, precipitate excessive synaptic elimination and ultimately compromise cognitive resilience.

The gut–immune axis provides a systemic influence on ageing: Butyrate exerts its beneficial effects through the inhibition of histone deacetylases (HDACs), which lead to changes in gene expression that promote an anti‐inflammatory and tissue‐protective phenotype. By inhibiting HDAC activity, butyrate increases histone acetylation, facilitating the transcription of genes such as those encoding TGF‐β1, a factor involved in tissue repair and immune tolerance (Martin‐Gallausiaux et al. 2018). This epigenetic reprogramming is considered an important process in counteracting the molecular drivers of ageing and reducing inflammaging, a chronic inflammatory state associated with the ageing process and its associated diseases (Chen and Vitetta 2020; Ferrucci and Fabbri 2018).

Among the research gaps due to the novelty of the topic, there is a critical need to distinguish between correlation and causation, and to conduct more studies with a longitudinal design. Many of the studies included are cross‐sectional, often comparing different individuals at various life stages rather than following the same individuals over time. While the life‐course approach adopted by the WHO provides a promising framework to understand ageing and intrinsic capacity, there is currently insufficient longitudinal data to establish causal links definitively. For example, although early‐life gut dysbiosis may influence later health outcomes, it cannot yet be assumed that individuals with such microbial imbalances in childhood will necessarily experience reduced vitality or diminished muscle mass in old age. A supportive environment in later life (e.g., a healthy lifestyle) can compensate for a dysbiotic state‐driven loss of intrinsic capacity domains in early life. Furthermore, it is evident that certain domains of intrinsic capacity and developmental stages are less explored than others concerning their relationship with gut microbiota. Several studies have delved into how gut microbiota and SCFAs influence locomotion, vitality and cognition. Although starting to emerge given the gut–brain connection, more research is needed for the psychological domain, in particular, to understand how gut health may affect energy levels, mood and stress. Even more evident is the under‐exploration of sensory capacities (i.e., hearing and vision domains) in this field. At the same time, the level of knowledge regarding gut microbiota in adolescents is generally less developed than in infants and adults. This difference in knowledge could be attributed to adolescence being a period of significant physiological, psychological and social changes. These changes can introduce more variables and complexities into research activities, challenging the definition of the gut microbiota's net effects and roles.

As a way forward, we believe gut microbiota is a fascinating and promising aspect of our lives that warrants further exploration and understanding. Its age‐related evolution makes it of particular interest to identify variables potentially affecting the trajectories of ageing, particularly healthy ageing. A recent report provided information on several ongoing clinical trials investigating the effects of microbiome‐related interventions on various outcomes related to healthy ageing (Guarente et al. 2024). Among these interventions, we might find in the future a microbiome‐related solution that can be delivered in a wide range of contexts to improve health and prevent age‐associated diseases.

## Author Contributions

**Christoph Benner:** conceptualisation, methodology, investigation, writing including original draft and revisions. **Matteo Cesari:** conceptualisation, methodology, supervision, writing including critical review, inputs and editing. **Ritu Sadana:** initiation, conceptualisation, methodology, supervision, writing including critical review, inputs and editing.

## Conflicts of Interest

The authors declare no conflicts of interest.

## Data Availability

Data sharing not applicable to this article as no datasets were generated or analysed during the current study.
